# Bis(1-methyl­piperidinium) tetra­chlorido­cuprate(II)

**DOI:** 10.1107/S1600536811016382

**Published:** 2011-05-07

**Authors:** Bryan J. Reynolds, Marcus R. Bond

**Affiliations:** a Department of Chemistry, Southeast Missouri State University, Cape Girardeau, MO 63701, USA

## Abstract

The structure of the title compound, (C_6_H_14_N)_2_[CuCl_4_], consists of two inequivalent 1-methyl­piperidinium cations and a flattened tetra­hedral [CuCl_4_]^2−^ anion. Each organic cation exhibits a chair conformation with the methyl group in the equatorial position. They are segregated into alternating layers parallel to (100) and stacked along [100]. The first cation is arranged in parallel stacks in a herringbone pattern with rows of [CuCl_4_]^2−^ anions fitting between the stacks and with a Cl^−^ ion directed into the inter­ior of the layer. The second organic cation forms distorted *hcp* layers that separate the other organic cation/[CuCl_4_]^2−^ slabs. N—H⋯Cl hydrogen bonding between the cations and the anions consolidates the crystal packing.

## Related literature

For background to compounds with [CuCl_4_]^2−^ anions, see: Awwadi *et al.* (2007 ▶); Bloomquist *et al.* (1988 ▶); Ihara (2007 ▶); Nelson *et al.* (1979 ▶); Schneider *et al.* (2007 ▶); Willett (1991 ▶); Willett & Twamley (2007 ▶). For related structures, see: Fernandez *et al.* (1987 ▶); Parent *et al.* (2007 ▶); Nalla & Bond (2011 ▶). For comparison bond lengths and angles, see: Ladd & Palmer (1994 ▶).
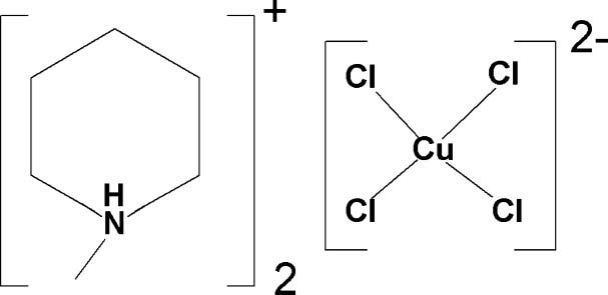


## Experimental

### 

#### Crystal data

(C_6_H_14_N)_2_[CuCl_4_]
*M* *_r_* = 405.7
Monoclinic, 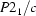

*a* = 12.2264 (2) Å
*b* = 11.3442 (2) Å
*c* = 13.3455 (2) Å
β = 96.865 (1)°
*V* = 1837.73 (5) Å^3^
*Z* = 4
Mo *K*α radiation
μ = 1.76 mm^−1^
*T* = 100 K
0.32 × 0.23 × 0.17 mm

#### Data collection

Nonius KappaCCD diffractometer
Absorption correction: multi-scan (*DENZO*/*SCALEPACK*; Otwinowski & Minor, 1997 ▶) *T*_min_ = 0.628, *T*_max_ = 0.694
16568 measured reflections
8453 independent reflections
6647 reflections with *I* > 2σ(*I*)
*R*_int_ = 0.026

#### Refinement

*R*[*F* ^2^ > 2σ(*F* ^2^)] = 0.030
*wR*(*F* ^2^) = 0.067
*S* = 1.05
8453 reflections
285 parameters
All H-atom parameters refined
Δρ_max_ = 0.57 e Å^−3^
Δρ_min_ = −0.58 e Å^−3^

### 

Data collection: *COLLECT* (Hooft, 1998 ▶); cell refinement: *SCALEPACK* (Otwinowski & Minor, 1997 ▶); data reduction: *DENZO* (Otwinowski & Minor, 1997 ▶) and *SCALEPACK*; program(s) used to solve structure: *SIR92* (Altomare *et al.*, 1993 ▶); program(s) used to refine structure: *SHELXL97* (Sheldrick, 2008 ▶); molecular graphics: *ORTEP-3 for Windows* (Farrugia, 1997 ▶) and *ORTEPIII* (Burnett & Johnson, 1996 ▶); software used to prepare material for publication: *WinGX* (Farrugia, 1999 ▶).

## Supplementary Material

Crystal structure: contains datablocks global, I. DOI: 10.1107/S1600536811016382/wm2484sup1.cif

Structure factors: contains datablocks I. DOI: 10.1107/S1600536811016382/wm2484Isup2.hkl

Additional supplementary materials:  crystallographic information; 3D view; checkCIF report

## Figures and Tables

**Table:** 

Cu1—Cl1	2.2816 (3)
Cu1—Cl2	2.2351 (3)
Cu1—Cl3	2.2539 (3)
Cu1—Cl4	2.2475 (3)

**Table:** 

Cl1—Cu1—Cl2	100.615 (11)
Cl1—Cu1—Cl3	98.880 (12)
Cl1—Cu1—Cl4	128.967 (13)
Cl2—Cu1—Cl3	135.003 (13)
Cl2—Cu1—Cl4	100.840 (12)
Cl3—Cu1—Cl4	97.568 (12)

**Table 2:** Hydrogen-bond geometry (Å, °)

*D*—H⋯*A*	*D*—H	H⋯*A*	*D*⋯*A*	*D*—H⋯*A*
N11—H11⋯Cl1	0.895 (16)	2.331 (16)	3.188 (1)	160 (1)
N21—H21⋯Cl3	0.833 (17)	2.508 (16)	3.280 (1)	155 (1)
N21—H21⋯Cl4	0.833 (17)	2.821 (17)	3.364 (1)	125 (1)

**Fig. 1.**
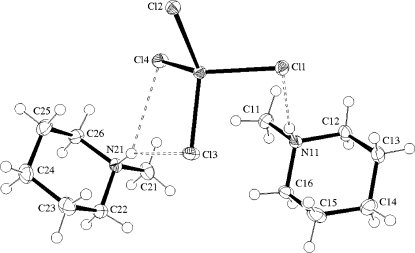
ORTEP diagram of the asymmetric unit with atom labels. Displacement ellipsoids are drawn at the 50% level. The hydrogen bonding between N—H and Cl is indicated with dotted lines.

**Fig. 2.**
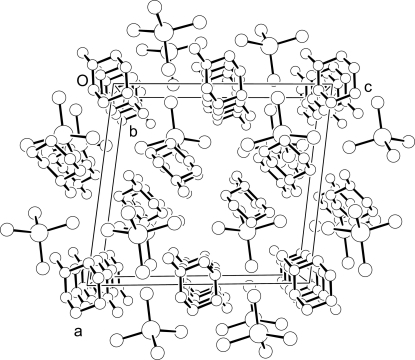
Unit cell packing diagram viewed along [010] showing the cation #1/[CuCl4]2- slabs at x = 0.0 and 1.0, and the cation #2 layer at x = 0.5. For clarity, hydrogen atoms are omitted and other atoms are drawn as circles of arbitrary radii ranked in size with Cu largest followed by Cl, then C and N smallest.

## supplementary crystallographic information

### Comment

[CuCl_4_]^2-^ anions exhibit geometries ranging from tetrahedral to square planar. The exact geometry adopted results from factors such as packing forces (Schneider *et al.*, 2007; Nelson *et al.*, 1979), hydrogen bonding (Bloomquist *et al.*, 1988; Willett and Twamley, 2007), and halide···halide interactions (Awwadi *et al.*, 2007), although the most commonly observed geometry is flattened tetrahedral with a *trans* Cl—Cu—Cl angle of ≈ 130–135° (Willett, 1991). A small number of thermochromic *A*_2_[CuCl_4_] compounds are known in which the low temperature (green-coloured) phase contains square-planar [CuCl_4_]^2-^ while the high-temperature (yellow) phase contains flattened tetrahedral [CuCl_4_]^2-^ anions (Ihara, 2007).

The crystal structure of the title compound, (1-methylpiperidinium)_2_CuCl_4_, is comprised of a [CuCl_4_]^2-^ anion and two symmetrically inequivalent 1-methylpiperidinium cations. The [CuCl_4_]^2-^ anion displays the typical flattened tetrahedral geometry with an average *trans* Cl—Cu—Cl angle of 131.99° at 100 K, so no thermochromic phase transition is observed upon cooling to at least this temperature. Each 1-methylpiperidinium ion exhibits a chair conformation with the methyl group equatorial. Bond lengths and angles for the inorganic complex and organic cation conform to expected values (Ladd & Palmer, 1994). An *ORTEP* diagram of the asymmetric unit is presented in Figure 1.

The two inequivalent organic cations are segregated into alternating layers parallel to (100) and stacked along [100]. The cation #1 layer (containing N11) arranges cations in parallel stacks along [010] and in a herringbone pattern with the cation plane approximately perpendicular to the layer plane (mean plane normal forming an angle of 91.63 (3)° with respect to a). Neighboring cations in the stacks are related by inversion and neighboring stacks are related by the *c*-glide plane. The [CuCl_4_]^2-^ anions form rows of translationally equivalent complexes parallel to [010] that fit in between the stacks and on both sides of the cation #1 layer. Atom Cl1 is directed almost into the middle of the cation layer with the rows of complexes on either side of the layer offset from one another to provide spacing between the Cl1 atoms inside the layer. The closest intermolecular Cl···Cl contact distance is, thus, Cl1···Cl1^i^= 5.891 (4) Å (i=-*x*, 1/2+*y*, 1/2-*z*) between Cl1 atoms from opposite rows of complexes. Cation #2 forms a layer at *x* = 0.5 to separate the cation #1/[CuCl_4_]^2-^ slabs at *x* = 0.0 and 1.0. In this layer the mean plane of the cation is closer to the layer plane with the normal forming an angle of 41.34° with respect to a. The cations in this layer are arranged in a distorted *hcp* pattern with neighboring cations related by inversion, 2_1_ rotation, or *c*-glide plane operations. A short, direct N11—H11···Cl1 hydrogen bond locks the Cl1 atoms into the interior of the cation #1 layer while a longer, less direct, and bifurcated N21—H21···Cl3 and ···Cl4 hydrogen bond links the cation #1/[CuCl_4_]^2-^ slabs to the cation #2 layer. A packing diagram for the structure viewed along [010] is presented in Figure 2.

In contrast to the layer structure of (1-methylpiperidinium)_2_[CuCl_4_], in the related piperdinium salt (Fernandez *et al.*, 1987; CSD refcode: PNCLCU01) translationally equivalent rows of [CuCl_4_]^2-^ anions are surrounded by stacks of translationally equivalent organic cations. N—H···Cl hydrogen bonding from one inequivalent organic cation links neighboring complexes in the same row while hydrogen bonding from the other inequivalent organic cation links complexes in different rows. So there are four direct hydrogen bonds in the piperidinium salt, *versus* one direct and one bifurcated in the title structure. Yet in spite of the greater degree of hydrogen bonding the average *trans* Cl—Cu—Cl angle of 133.49° (at room temperature) is only slightly larger. (1-Methylmorpholinium)_2_[CuCl_4_] (Parent *et al.*, 2007; CSD refcode: VICMIE) also has rows or chains of [CuCl_4_]^2-^ anions with short Cl···Cl contacts surrounded by parallel stacks of organic cations. The two inequivalent organic cations each form a bifurcated hydrogen bond to all four Cl ligands of the complex, which has an average *trans* Cl—Cu—Cl angle of 134.32° (158 K). The *N*,*N*-dimethylpiperidinium system has been studied, although no examples of a [CuCl_4_]^2-^ salt have yet been found. Crystals of a light green [CuCl_3_(H_2_O)]^-^ salt do, however, form readily (Nalla and Bond, 2011).

### Experimental

5 ml of 1-methylpiperidine were neutralized with concentrated HCl. This solution was mixed in a 2:1 molar ratio with copper(II) chloride dissolved in 6*M* HCl. Slow evaporation yielded yellow crystals of the title compound.

### Refinement

All non-hydrogen atoms were refined with anisotropic displacement parameters. All hydrogen atoms were visible on electron density difference maps and freely refined to give N—H = 0.833–0.895 Å, methylene C—H = 0.913–1.03 Å. and methyl C—H = 0.945–0.97 Å.

### Crystal data

**Table:**

(C_6_H_14_N)_2_[CuCl_4_]	*F*(000) = 844
*M**_r_* = 405.7	*D*_x_ = 1.466 Mg m^−^^3^
Monoclinic, *P*2_1_/*c*	Mo *K*α radiation, λ = 0.71073 Å
*a* = 12.2264 (2) Å	Cell parameters from 8818 reflections
*b* = 11.3442 (2) Å	θ = 1.0–35.6°
*c* = 13.3455 (2) Å	µ = 1.76 mm^−^^1^
β = 96.865 (1)°	*T* = 100 K
*V* = 1837.73 (5) Å^3^	Block, yellow
*Z* = 4	0.32 × 0.23 × 0.17 mm

### Data collection

**Table:**

Nonius KappaCCD diffractometer	8453 independent reflections
graphite	6647 reflections with *I* > 2σ(*I*)
Detector resolution: 9 pixels mm^-1^	*R*_int_ = 0.026
ω and φ scans	θ_max_ = 35.6°, θ_min_ = 4.0°
Absorption correction: multi-scan (*DENZO*/*SCALEPACK*; Otwinowski & Minor, 1997)	*h* = −19→20
*T*_min_ = 0.628, *T*_max_ = 0.694	*k* = −18→18
16568 measured reflections	*l* = −21→21

### Refinement

**Table:**

Refinement on *F*^2^	Secondary atom site location: difference Fourier map
Least-squares matrix: full	Hydrogen site location: difference Fourier map
*R*[*F*^2^ > 2σ(*F*^2^)] = 0.030	All H-atom parameters refined
*wR*(*F*^2^) = 0.067	*w* = 1/[σ^2^(*F*_o_^2^) + (0.0249*P*)^2^ + 0.6467*P*] where *P* = (*F*_o_^2^ + 2*F*_c_^2^)/3
*S* = 1.05	(Δ/σ)_max_ = 0.006
8453 reflections	Δρ_max_ = 0.57 e Å^−^^3^
285 parameters	Δρ_min_ = −0.58 e Å^−^^3^
0 restraints	Extinction correction: *SHELXL97* (Sheldrick, 2008), Fc^*^=kFc[1+0.001xFc^2^λ^3^/sin(2θ)]^-1/4^
Primary atom site location: structure-invariant direct methods	Extinction coefficient: 0.0017 (3)

### Special details

**Table:**

Geometry. All e.s.d.'s (except the e.s.d. in the dihedral angle between two l.s. planes) are estimated using the full covariance matrix. The cell e.s.d.'s are taken into account individually in the estimation of e.s.d.'s in distances, angles and torsion angles; correlations between e.s.d.'s in cell parameters are only used when they are defined by crystal symmetry. An approximate (isotropic) treatment of cell e.s.d.'s is used for estimating e.s.d.'s involving l.s. planes.
Refinement. Refinement of *F*^2^ against ALL reflections. The weighted *R*-factor *wR* and goodness of fit *S* are based on *F*^2^, conventional *R*-factors *R* are based on *F*, with *F* set to zero for negative *F*^2^. The threshold expression of *F*^2^ > σ(*F*^2^) is used only for calculating *R*-factors(gt) *etc*. and is not relevant to the choice of reflections for refinement. *R*-factors based on *F*^2^ are statistically about twice as large as those based on *F*, and *R*- factors based on ALL data will be even larger.

### Fractional atomic coordinates and isotropic or equivalent isotropic displacement parameters (Å^2^)

**Table:**

	*x*	*y*	*z*	*U*_iso_*/*U*_eq_	
Cu1	0.251677 (12)	0.415201 (12)	0.299141 (10)	0.01376 (4)	
Cl1	0.06546 (2)	0.42579 (2)	0.25734 (2)	0.01674 (5)	
Cl2	0.27180 (2)	0.52180 (2)	0.44100 (2)	0.01610 (5)	
Cl3	0.30403 (3)	0.43621 (3)	0.14382 (2)	0.02020 (6)	
Cl4	0.35629 (3)	0.25748 (3)	0.34713 (2)	0.01954 (6)	
N11	0.07370 (8)	0.22960 (9)	0.08737 (7)	0.01501 (17)	
H11	0.0798 (13)	0.2956 (15)	0.1244 (12)	0.020 (4)*	
C11	0.14106 (11)	0.13720 (12)	0.14522 (10)	0.0219 (2)	
H11A	0.1456 (15)	0.0723 (16)	0.1014 (13)	0.028 (5)*	
H11B	0.2116 (14)	0.1704 (15)	0.1677 (12)	0.024 (4)*	
H11C	0.1045 (16)	0.1151 (17)	0.2025 (15)	0.037 (5)*	
C12	−0.04575 (10)	0.19611 (12)	0.07114 (10)	0.0206 (2)	
H12A	−0.0483 (14)	0.1249 (16)	0.0391 (12)	0.023 (4)*	
H12B	−0.0671 (14)	0.1811 (15)	0.1347 (13)	0.024 (4)*	
C13	−0.11317 (11)	0.29197 (12)	0.01392 (10)	0.0211 (2)	
H13A	−0.1880 (15)	0.2688 (15)	0.0042 (13)	0.026 (4)*	
H13B	−0.1108 (14)	0.3600 (16)	0.0564 (13)	0.026 (4)*	
C14	−0.07016 (12)	0.32027 (14)	−0.08588 (10)	0.0264 (3)	
H14A	−0.0764 (15)	0.2522 (17)	−0.1302 (14)	0.033 (5)*	
H14B	−0.1129 (15)	0.3849 (17)	−0.1173 (14)	0.033 (5)*	
C15	0.05150 (12)	0.35232 (14)	−0.06641 (11)	0.0275 (3)	
H15A	0.0813 (15)	0.3663 (17)	−0.1260 (14)	0.031 (5)*	
H15B	0.0616 (17)	0.4262 (18)	−0.0268 (15)	0.040 (5)*	
C16	0.11738 (11)	0.25462 (11)	−0.01073 (9)	0.0190 (2)	
H16A	0.1110 (13)	0.1833 (14)	−0.0472 (11)	0.017 (4)*	
H16B	0.1918 (14)	0.2742 (14)	0.0066 (12)	0.019 (4)*	
N21	0.51192 (8)	0.25190 (9)	0.15565 (8)	0.01564 (18)	
H21	0.4620 (13)	0.2945 (14)	0.1731 (12)	0.017 (4)*	
C21	0.45664 (12)	0.14160 (12)	0.11569 (11)	0.0232 (2)	
H21A	0.5108 (15)	0.0891 (16)	0.0926 (14)	0.030 (5)*	
H21B	0.4240 (16)	0.1038 (17)	0.1704 (15)	0.036 (5)*	
H21C	0.4060 (14)	0.1605 (16)	0.0588 (13)	0.028 (4)*	
C22	0.55885 (11)	0.31840 (12)	0.07359 (9)	0.0203 (2)	
H22A	0.6091 (13)	0.2664 (14)	0.0498 (12)	0.020 (4)*	
H22B	0.4995 (15)	0.3324 (16)	0.0233 (13)	0.027 (4)*	
C23	0.61029 (13)	0.43323 (12)	0.11370 (11)	0.0259 (3)	
H23A	0.6408 (16)	0.4715 (17)	0.0596 (14)	0.034 (5)*	
H23B	0.5515 (15)	0.4840 (16)	0.1296 (13)	0.030 (5)*	
C24	0.69579 (12)	0.41383 (14)	0.20480 (11)	0.0276 (3)	
H24A	0.7614 (16)	0.3662 (18)	0.1844 (14)	0.039 (5)*	
H24B	0.7199 (17)	0.491 (2)	0.2345 (15)	0.045 (6)*	
C25	0.64709 (12)	0.34239 (14)	0.28531 (10)	0.0256 (3)	
H25A	0.7000 (15)	0.3232 (16)	0.3397 (13)	0.030 (5)*	
H25B	0.5928 (15)	0.3843 (16)	0.3125 (13)	0.025 (4)*	
C26	0.59873 (11)	0.22741 (12)	0.24265 (9)	0.0206 (2)	
H26A	0.6515 (13)	0.1796 (14)	0.2156 (11)	0.019 (4)*	
H26B	0.5624 (13)	0.1821 (14)	0.2901 (12)	0.018 (4)*	

### Atomic displacement parameters (Å^2^)

**Table:**

	*U*^11^	*U*^22^	*U*^33^	*U*^12^	*U*^13^	*U*^23^
Cu1	0.01396 (7)	0.01435 (7)	0.01306 (6)	0.00173 (5)	0.00206 (4)	−0.00012 (5)
Cl1	0.01474 (12)	0.01853 (12)	0.01674 (11)	0.00235 (9)	0.00100 (9)	−0.00114 (9)
Cl2	0.01717 (12)	0.01518 (12)	0.01603 (11)	−0.00021 (9)	0.00233 (9)	−0.00179 (9)
Cl3	0.02281 (14)	0.02322 (14)	0.01554 (12)	0.00504 (11)	0.00628 (10)	0.00299 (10)
Cl4	0.02184 (14)	0.01968 (13)	0.01715 (12)	0.00794 (10)	0.00254 (10)	0.00137 (9)
N11	0.0150 (4)	0.0163 (4)	0.0139 (4)	−0.0004 (3)	0.0024 (3)	−0.0013 (3)
C11	0.0210 (6)	0.0241 (6)	0.0199 (5)	0.0035 (5)	−0.0001 (4)	0.0035 (5)
C12	0.0159 (5)	0.0209 (6)	0.0249 (6)	−0.0028 (4)	0.0024 (4)	0.0056 (5)
C13	0.0153 (5)	0.0222 (6)	0.0259 (6)	0.0016 (4)	0.0030 (4)	0.0026 (5)
C14	0.0264 (7)	0.0306 (7)	0.0219 (6)	0.0090 (6)	0.0013 (5)	0.0068 (5)
C15	0.0279 (7)	0.0290 (7)	0.0278 (6)	0.0073 (6)	0.0127 (5)	0.0135 (5)
C16	0.0194 (6)	0.0216 (6)	0.0171 (5)	0.0023 (4)	0.0067 (4)	0.0005 (4)
N21	0.0142 (4)	0.0144 (4)	0.0184 (4)	0.0005 (3)	0.0022 (3)	−0.0018 (3)
C21	0.0212 (6)	0.0185 (6)	0.0307 (7)	−0.0037 (5)	0.0059 (5)	−0.0071 (5)
C22	0.0211 (6)	0.0225 (6)	0.0174 (5)	−0.0028 (5)	0.0024 (4)	0.0005 (4)
C23	0.0281 (7)	0.0207 (6)	0.0295 (7)	−0.0066 (5)	0.0062 (5)	0.0004 (5)
C24	0.0211 (6)	0.0306 (7)	0.0312 (7)	−0.0085 (5)	0.0031 (5)	−0.0106 (6)
C25	0.0223 (6)	0.0334 (7)	0.0200 (6)	0.0009 (5)	−0.0009 (5)	−0.0086 (5)
C26	0.0202 (6)	0.0228 (6)	0.0185 (5)	0.0056 (5)	0.0014 (4)	0.0001 (4)

### Geometric parameters (Å, °)

**Table:**

Cu1—Cl1	2.2816 (3)	C16—H16A	0.943 (16)
Cu1—Cl2	2.2351 (3)	C16—H16B	0.938 (16)
Cu1—Cl3	2.2539 (3)	N21—C21	1.4903 (16)
Cu1—Cl4	2.2475 (3)	N21—C22	1.4992 (16)
N11—C11	1.4904 (16)	N21—C26	1.5024 (16)
N11—C16	1.4990 (15)	N21—H21	0.833 (16)
N11—C12	1.4995 (16)	C21—H21A	0.968 (19)
N11—H11	0.895 (17)	C21—H21B	0.97 (2)
C11—H11A	0.946 (18)	C21—H21C	0.945 (18)
C11—H11B	0.957 (17)	C22—C23	1.5160 (19)
C11—H11C	0.96 (2)	C22—H22A	0.934 (17)
C12—C13	1.5148 (18)	C22—H22B	0.941 (18)
C12—H12A	0.913 (18)	C23—C24	1.522 (2)
C12—H12B	0.932 (17)	C23—H23A	0.955 (19)
C13—C14	1.5233 (19)	C23—H23B	0.964 (19)
C13—H13A	0.946 (18)	C24—C25	1.523 (2)
C13—H13B	0.956 (18)	C24—H24A	1.03 (2)
C14—C15	1.523 (2)	C24—H24B	0.99 (2)
C14—H14A	0.970 (19)	C25—C26	1.515 (2)
C14—H14B	0.966 (19)	C25—H25A	0.938 (18)
C15—C16	1.5121 (19)	C25—H25B	0.925 (18)
C15—H15A	0.927 (18)	C26—H26A	0.948 (16)
C15—H15B	0.99 (2)	C26—H26B	0.964 (16)
			
Cl1—Cu1—Cl2	100.615 (11)	N11—C16—H16B	105.4 (10)
Cl1—Cu1—Cl3	98.880 (12)	C15—C16—H16B	113.0 (10)
Cl1—Cu1—Cl4	128.967 (13)	H16A—C16—H16B	110.4 (13)
Cl2—Cu1—Cl3	135.003 (13)	C21—N21—C22	110.98 (10)
Cl2—Cu1—Cl4	100.840 (12)	C21—N21—C26	111.69 (10)
Cl3—Cu1—Cl4	97.568 (12)	C22—N21—C26	111.15 (10)
C11—N11—C16	110.68 (10)	C21—N21—H21	105.7 (11)
C11—N11—C12	111.41 (10)	C22—N21—H21	105.7 (11)
C16—N11—C12	111.37 (10)	C26—N21—H21	111.4 (11)
C11—N11—H11	107.3 (10)	N21—C21—H21A	109.4 (11)
C16—N11—H11	108.0 (10)	N21—C21—H21B	108.1 (11)
C12—N11—H11	107.9 (10)	H21A—C21—H21B	109.0 (15)
N11—C11—H11A	107.3 (11)	N21—C21—H21C	108.8 (11)
N11—C11—H11B	107.9 (10)	H21A—C21—H21C	106.9 (15)
H11A—C11—H11B	112.6 (15)	H21B—C21—H21C	114.5 (16)
N11—C11—H11C	108.3 (12)	N21—C22—C23	110.69 (10)
H11A—C11—H11C	110.8 (16)	N21—C22—H22A	104.8 (10)
H11B—C11—H11C	109.9 (15)	C23—C22—H22A	113.6 (10)
N11—C12—C13	110.66 (10)	N21—C22—H22B	106.0 (11)
N11—C12—H12A	105.7 (11)	C23—C22—H22B	111.0 (11)
C13—C12—H12A	114.4 (11)	H22A—C22—H22B	110.3 (14)
N11—C12—H12B	106.7 (10)	C22—C23—C24	111.97 (12)
C13—C12—H12B	113.3 (10)	C22—C23—H23A	107.8 (11)
H12A—C12—H12B	105.4 (15)	C24—C23—H23A	112.2 (11)
C12—C13—C14	111.60 (11)	C22—C23—H23B	107.6 (11)
C12—C13—H13A	109.2 (10)	C24—C23—H23B	111.7 (10)
C14—C13—H13A	111.6 (10)	H23A—C23—H23B	105.3 (15)
C12—C13—H13B	107.7 (10)	C23—C24—C25	110.59 (12)
C14—C13—H13B	111.1 (10)	C23—C24—H24A	110.3 (11)
H13A—C13—H13B	105.4 (14)	C25—C24—H24A	106.9 (11)
C15—C14—C13	109.47 (11)	C23—C24—H24B	109.7 (12)
C15—C14—H14A	107.4 (11)	C25—C24—H24B	108.0 (12)
C13—C14—H14A	110.8 (11)	H24A—C24—H24B	111.3 (16)
C15—C14—H14B	111.1 (11)	C26—C25—C24	111.17 (11)
C13—C14—H14B	108.3 (11)	C26—C25—H25A	107.1 (11)
H14A—C14—H14B	109.9 (15)	C24—C25—H25A	112.2 (11)
C16—C15—C14	111.15 (12)	C26—C25—H25B	108.9 (11)
C16—C15—H15A	107.8 (12)	C24—C25—H25B	111.3 (11)
C14—C15—H15A	111.8 (11)	H25A—C25—H25B	106.1 (15)
C16—C15—H15B	109.5 (12)	N21—C26—C25	109.82 (11)
C14—C15—H15B	110.5 (12)	N21—C26—H26A	105.2 (9)
H15A—C15—H15B	106.0 (16)	C25—C26—H26A	112.5 (10)
N11—C16—C15	110.09 (10)	N21—C26—H26B	105.5 (9)
N11—C16—H16A	105.8 (9)	C25—C26—H26B	113.7 (9)
C15—C16—H16A	111.7 (9)	H26A—C26—H26B	109.5 (13)

### Hydrogen-bond geometry (Å, °)

**Table:**

*D*—H···*A*	*D*—H	H···*A*	*D*···*A*	*D*—H···*A*
N11—H11···Cl1	0.895 (16)	2.331 (16)	3.188 (1)	160 (1)
N21—H21···Cl3	0.833 (17)	2.508 (16)	3.280 (1)	155 (1)
N21—H21···Cl4	0.833 (17)	2.821 (17)	3.364 (1)	125 (1)
